# Supplementary material for: Millimeter-sized smart sensors reveal that a solar refuge protects tree snail Partula hyalina from extirpation
Source: Commun Biol. 2021 Jun 15;4:744. doi: 10.1038/s42003-021-02124-y (PMC8206136; doi:10.1038/s42003-021-02124-y)
Supplement: Supplementary file 2 — Supplementary Information [file 42003_2021_2124_MOESM2_ESM.pdf]

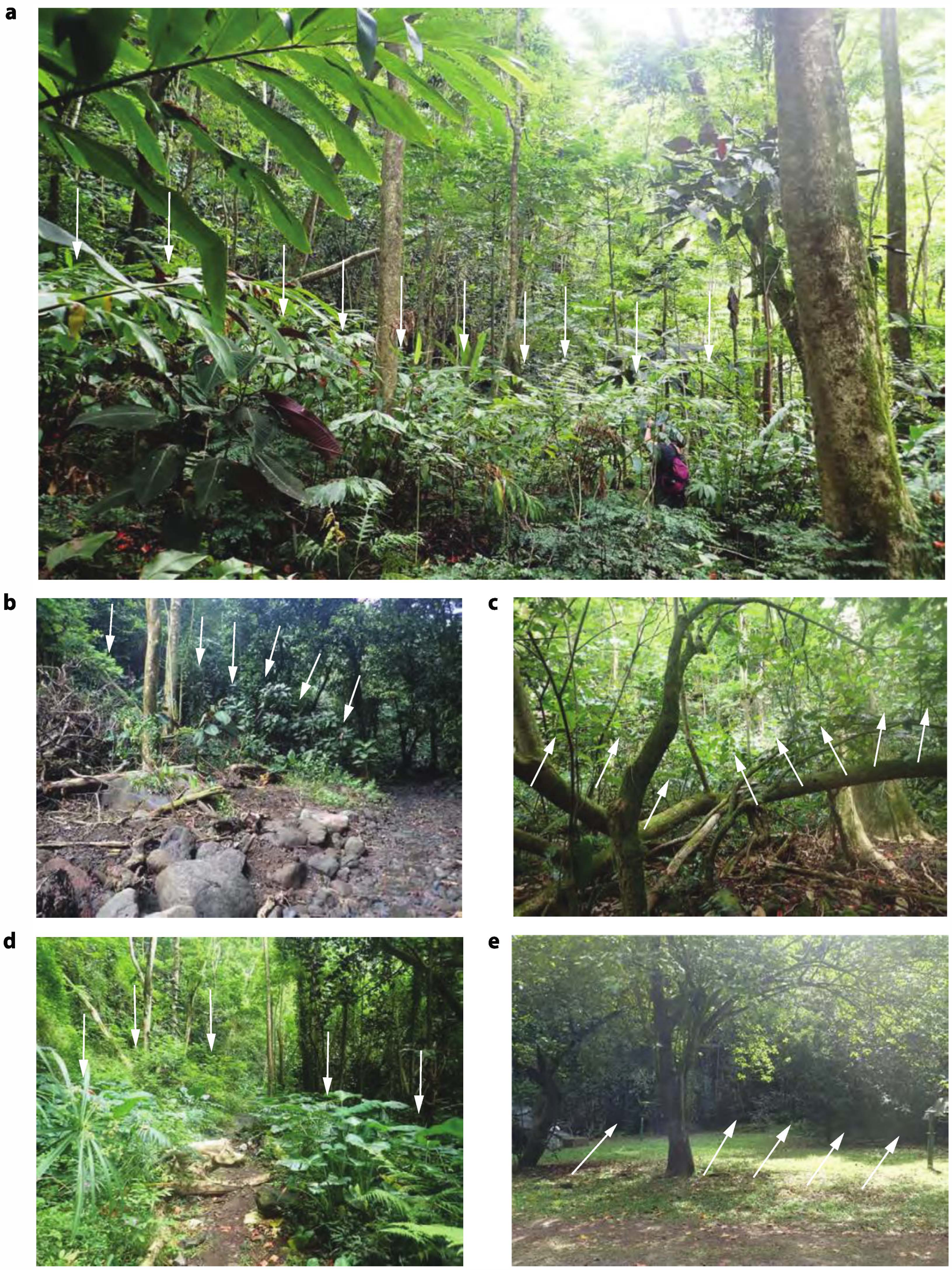

Supplementary Figure 1a-e. Representative views of the 5 Tahitian field locations used in this study. **a**, View of the Tipaerui-Iti Valley study site showing portion of the linear *Etlingera cevuga* stand (arrows) where the aestivating *Partula hyalina* specimens had their solar ecologies characterized. **b, c,** respectively views of the Faarapa Valley and Matatia Valley sites with arrows again pointing out the location of the aestivating specimens of *P. hyalina* specimens investigated. **d,** the Fautaua-Iti Valley site consisting of an open trail through the rainforest. Arrows point to the vegetation on either side of the trail where the predator (*Euglandina rosea*) specimens were located and released after sensor attachment. **e,** a representative portion of the Fautaua Valley study site, consisting of a forest edge adjoining an open grassy area. Arrows indicate the undergrowth just inside the forest where *E. rosea* specimens were caught and released.

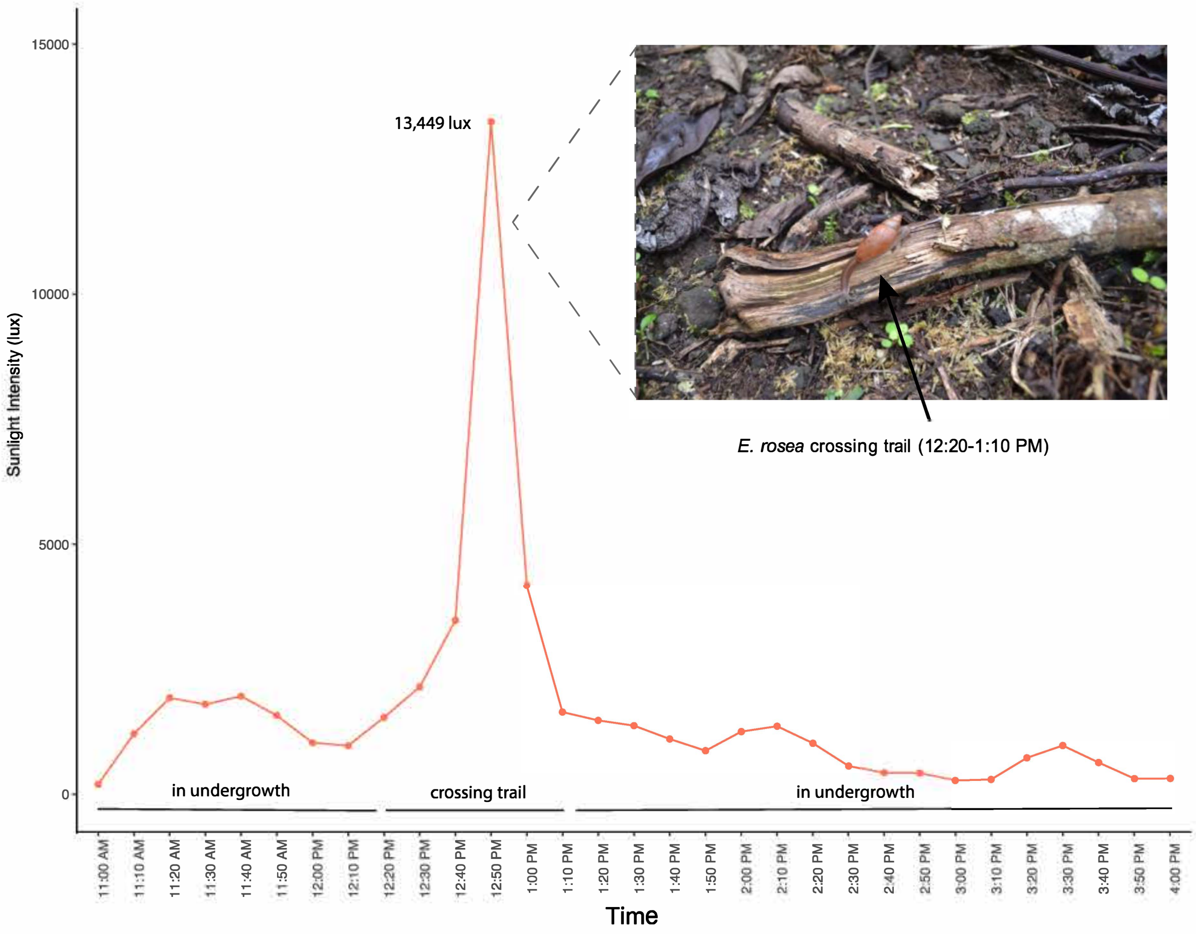

Supplementary Figure 2. August 11th 2017 solar ecology profile of a Fautaua-Iti Valley *Euglandina rosea* specimen. Note that it recorded by far the highest predator light intensity reading (13,449 lux) observed in this study at 12:50 pm when the overcast conditions briefly lifted while it was crossing the open trail that bisected the study site (Supplementary Figure 1d).

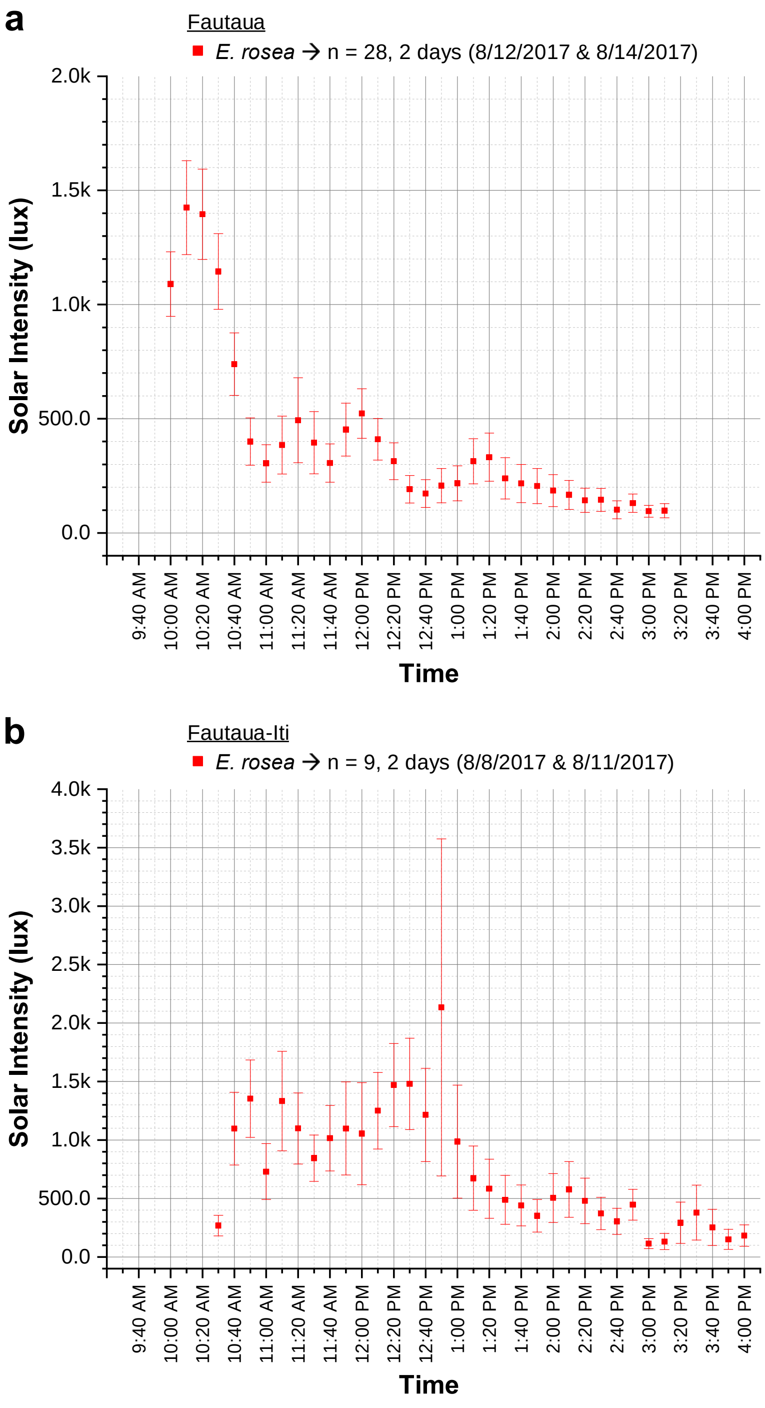

Supplementary Figure 3. a, Recorded daily solar ecology profiles, integrated over 10-minute intervals, for field specimens of *Euglandina rosea*. Fautaua Valley study site mean and standard errors of light sensor readings were obtained from a total of 28 *E. rosea* specimens over two days (August 12th and 14th, 2017). **b,** Fautaua-Iti Valley study site mean and standard errors of light sensor readings were obtained from a total of 9 *E. rosea* specimer two days (August 8th and 11th, 2017). The higher mean/variance values recorded for the 12:50 pm time interval stems primarily from a single snail that was briefly exposed to 13,449 lux - see Supplementary Figure 3.

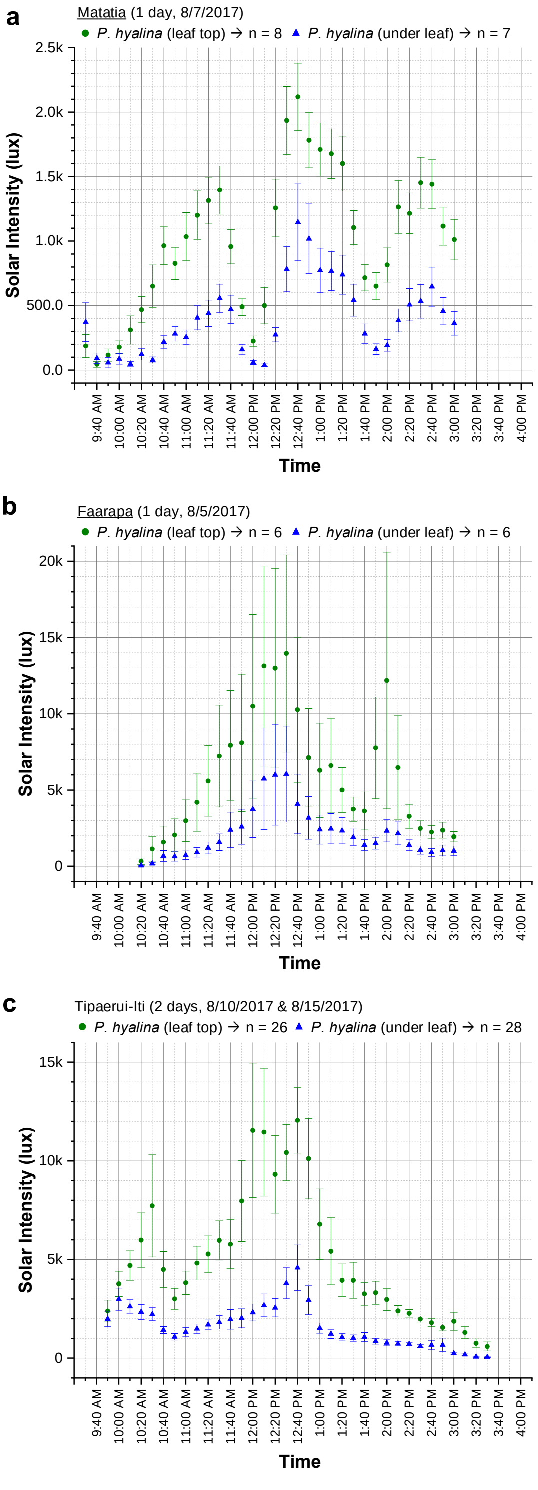

Supplementary Figure 4. a, Solar ecology profiles obtained for 8 aestivating Matatia Valley *Partula hyalina* tree snails on August 7th, 2017 (an overcast, rainy day). **b,** Solar ecology profiles obtained for 6 aestivating Faarapa Valley *Partula hyalina* tree snails on August 5th, 2017. **c,** Solar ecology profiles obtained for 28 aestivating Tipaerui-Iti Valley specimens of *Partula hyalina* tree snails over two days (August 10th and 15th, 2017). Sensor readings were intergrated over 10-minute time intervals and are presented as mean values with standard errors. Under leaf sensors recorded the tree snails immediate light environment whereas leaf top sensors recorded the ambient light environment of the supporting leaves.

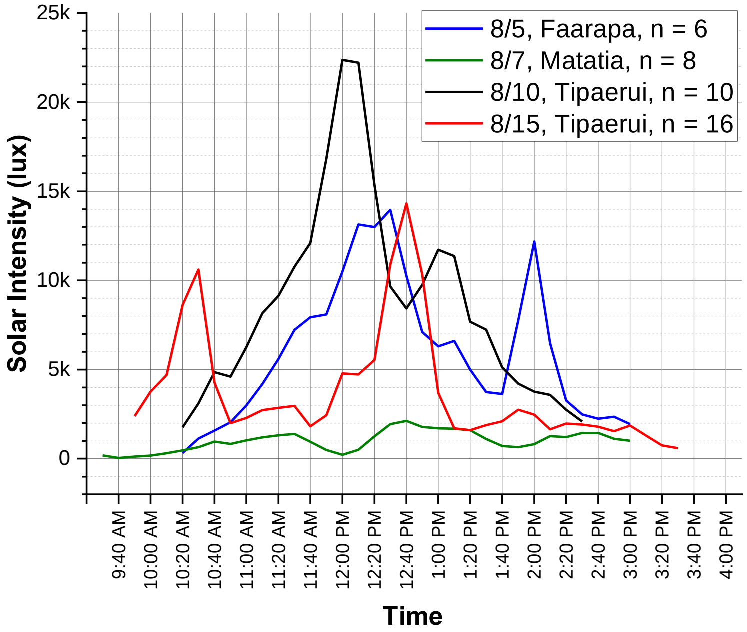

Supplementary Figure 5. Daily mean leaf top sensor light intensities recorded for aestivating field specimens of *Partula hyalina* over four days of recording. Sensor readings were integrated over 10-minute time intervals. Note the divergent August 10th and 15th solar irradiation profiles of leaves supporting Tipaerui-Iti Valley tree snails.

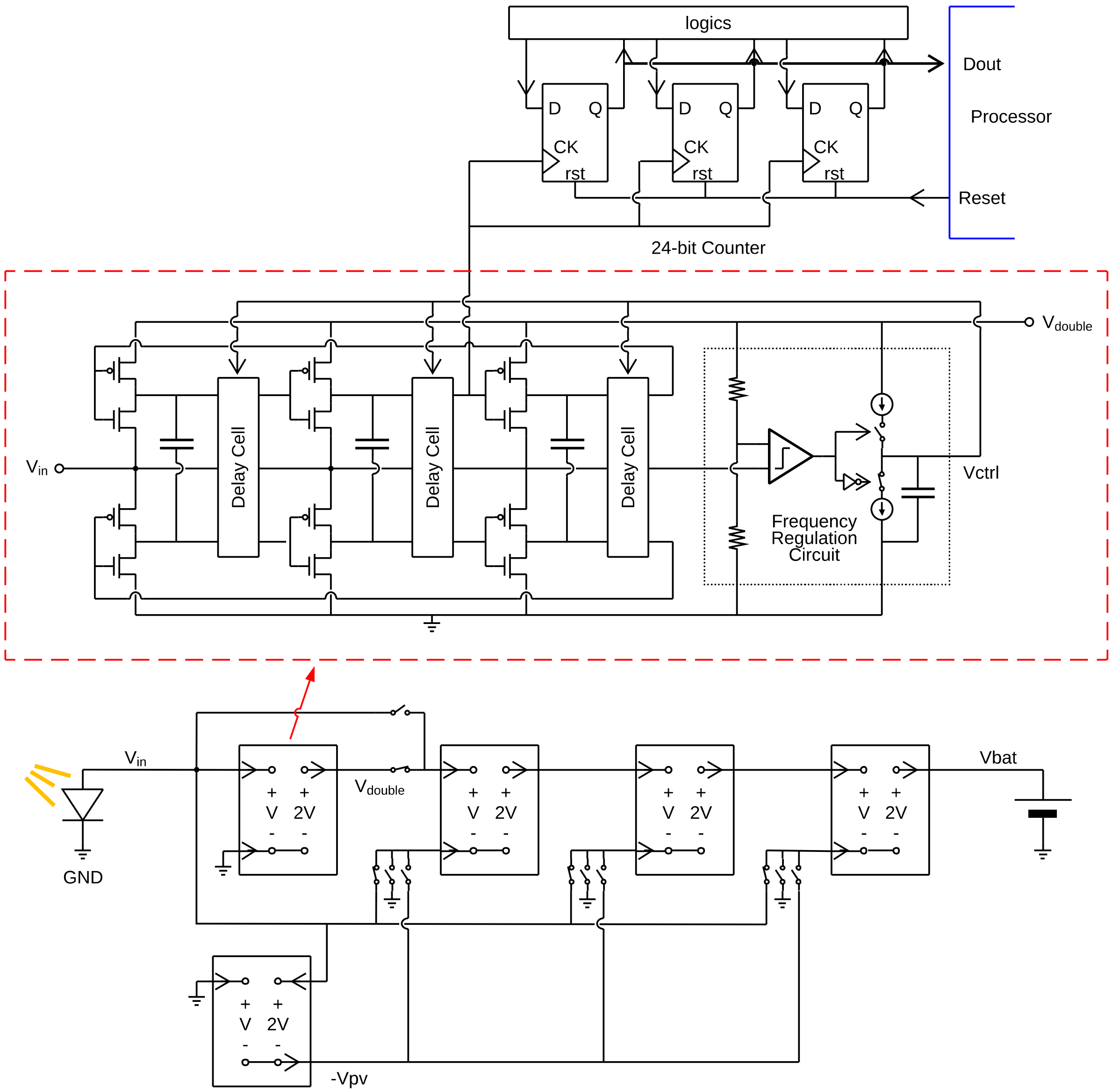

Supplementary Figure 6. Energy harvester and counter in the smart sensor for measuring solar ecology. Bottom shows the top level circuit diagram consisting of 5 voltage doubling circuits. Each doubler consists of a pair of coupled ring oscillators that are regulated in frequency such that they transfer a fixed charge each oscillation cycle (center circuit blow up). Oscillations are counted using the 24-bit counter shown at top of figure which gives a reading proportional to the light intensity.

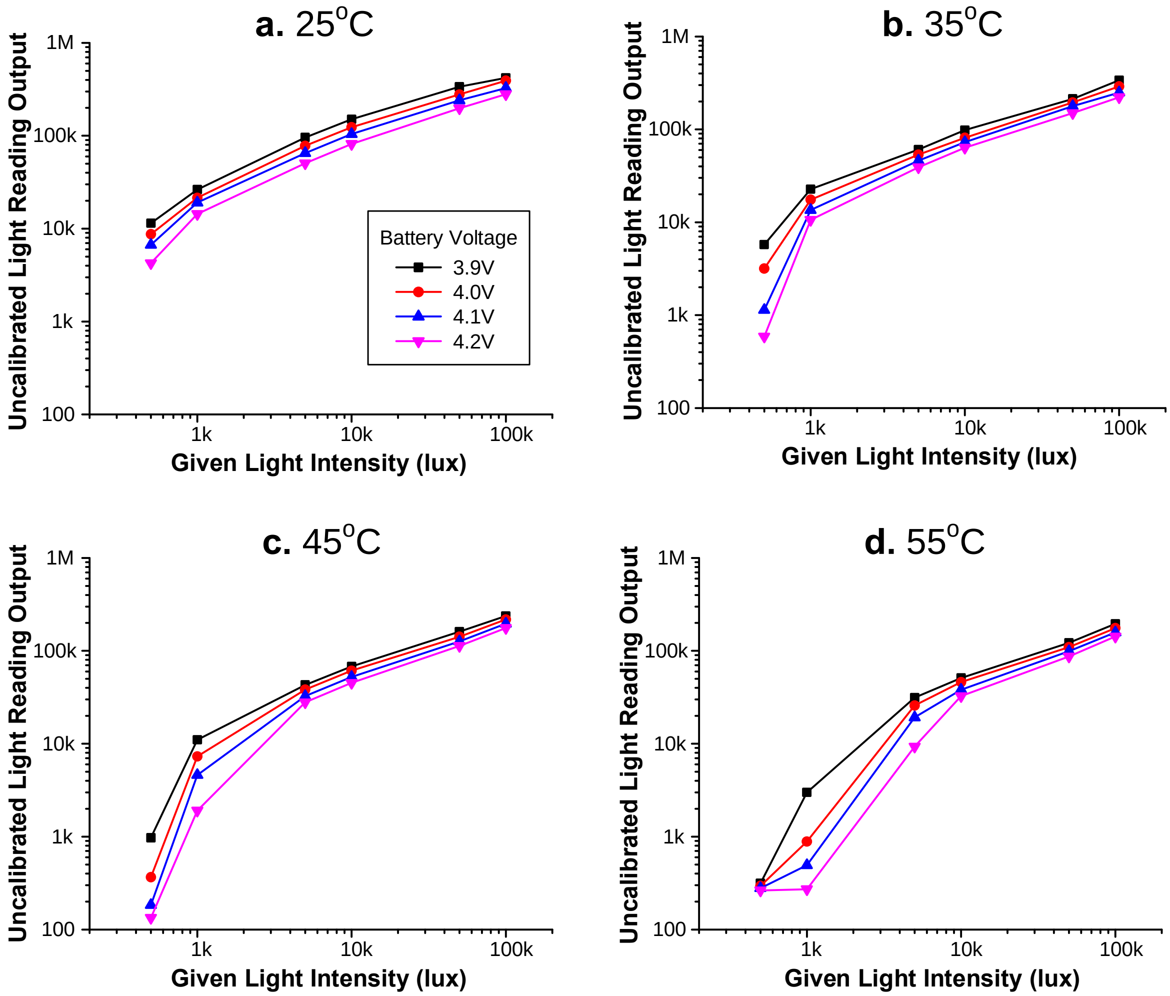

Supplementary Figure 7. Measured sensor data for construction of a piece-wise linear model of light intensity as a function of measure light reading output code, battery voltage and temperature. **a**, 25°C. **b**, 35°C. **c**, 45°C. **d**, 55°C. Light output codes were recorded for 4 smart sensor across 6 light conditions, 4 temperatures, and 4 battery voltages. The reading were averaged across the 4 sensor samples to create a piece-wise linear relationship between the four parameters (light level, voltage, temperature, output code). The model was then calibrated for each smart sensor.

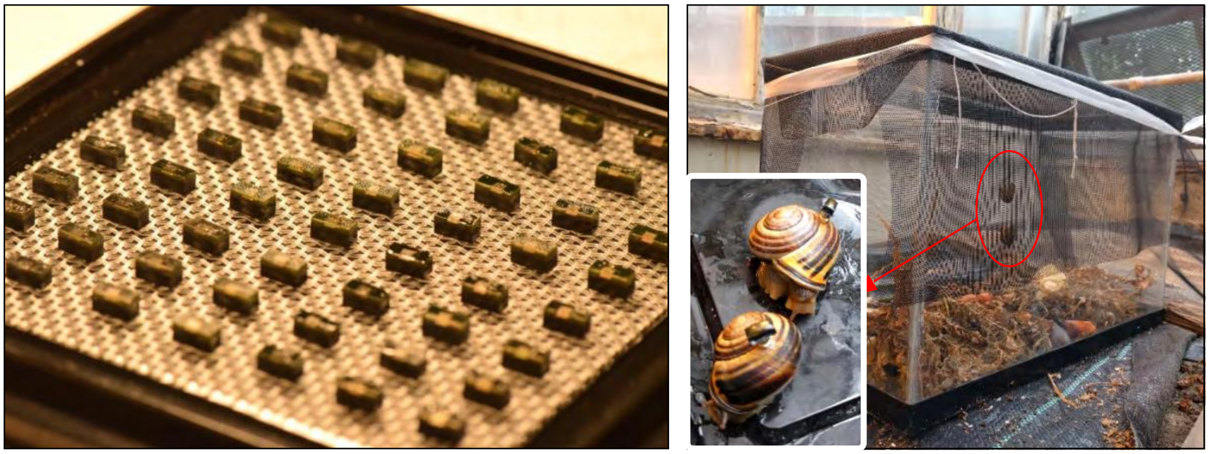

Supplementary Figure 8. Left: an array of 55 smart sensors readied for deployment in Tahiti. Each smart sensor is completely encapsulated with black and clear epoxy. Right: testing of smart sensors at the University of Michigan using locally caught *Cepaea nemoralis* land snails to confirm correct sensor operation and robust sensor attachment before deployment in Tahiti. Inset: Two specimens of *Cepaea nemoralis* with attached smart sensors.

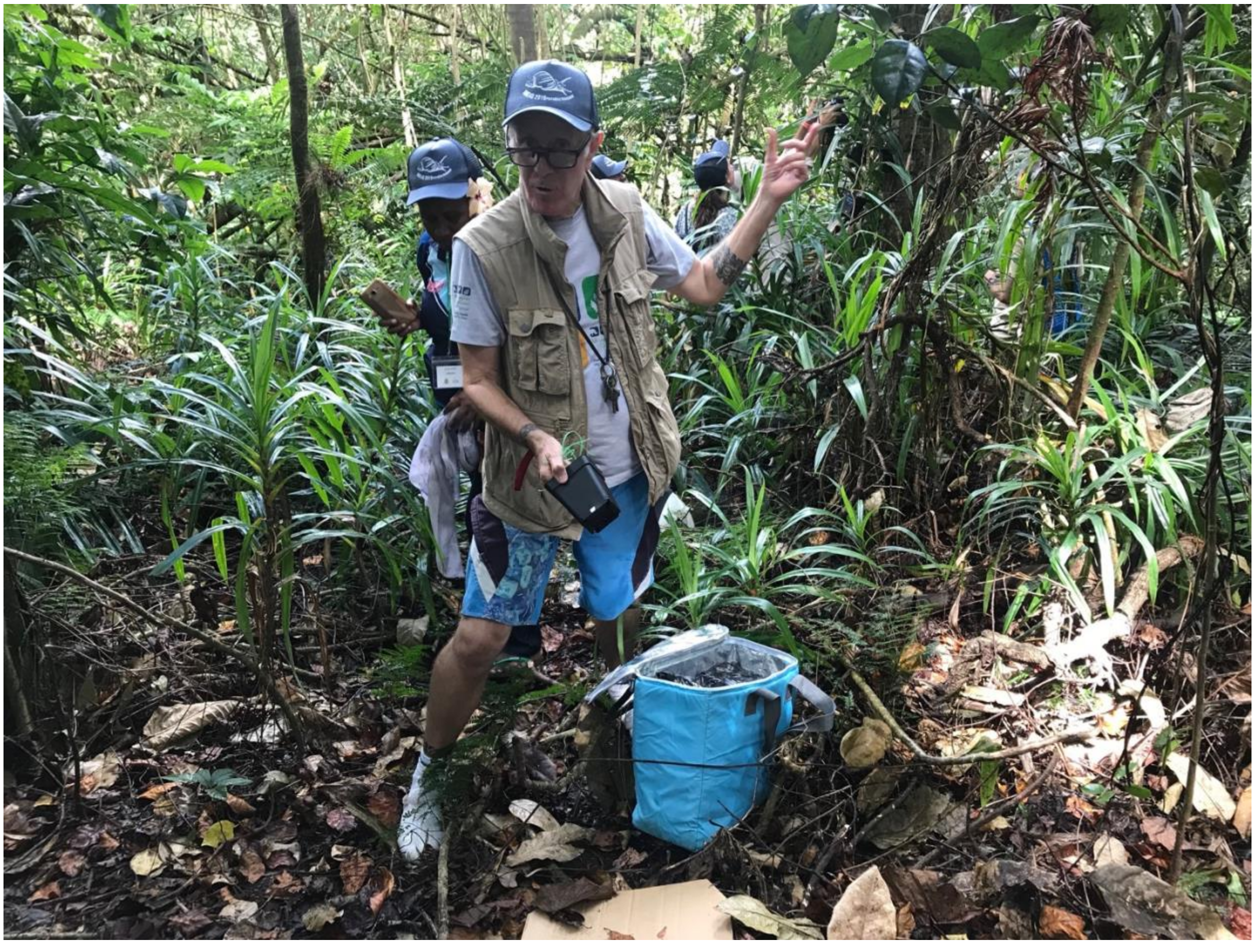

Supplementary Figure 9. Trevor Coote (foreground) directing the reintroduction of captive *Partula* tree snails (in the blue cooler carrying pack) to Moorea, August 27th 2019. The reintroduced snails were Moorean endemics that had been maintained in captivity for decades by the Partulid Global Species Management Programme, coordinated by the Zoological Society of London.
