# Supplementary material for: Millimeter-sized smart sensors reveal that a solar refuge protects tree snail Partula hyalina from extirpation
Source: Commun Biol. 2021 Jun 15;4:744. doi: 10.1038/s42003-021-02124-y (PMC8206136; doi:10.1038/s42003-021-02124-y)
Supplement: Supplementary file 3 — Description of Additional Supplementary Files [file 42003_2021_2124_MOESM3_ESM.pdf]

Description of Additional Supplementary Files

**File name:** Supplementary Movie 1

**Description:** Video recording of foraging Fautaua Valley (Tahiti) Euglandina rosea specimen with an attached smart sensor on its shell, taken August 14, 2017: https://www.youtube.com/watch?v=WEWSbk031QE.&feature=youtu.be Also available in the figshare depository (https://figshare.com/s/5958b330d9ae094db4c8).
