# Supplementary material for: Millimeter-sized smart sensors reveal that a solar refuge protects tree snail Partula hyalina from extirpation
Source: Commun Biol. 2021 Jun 15;4:744. doi: 10.1038/s42003-021-02124-y (PMC8206136; doi:10.1038/s42003-021-02124-y)
Supplement: Supplementary file 5 — Reporting Summary [file 42003_2021_2124_MOESM5_ESM.pdf]

nature research

Corresponding author(s): Diarmaid Ó Foighil

Last updated by author(s): Mar 4, 2021

Reporting Summary

Nature Research wishes to improve the reproducibility of the work that we publish. This form provides structure for consistency and transparency in reporting. For further information on Nature Research policies, see our Editorial Policies and the Editorial Policy Checklist.

Statistics

For all statistical analyses, confirm that the following items are present in the figure legend, table legend, main text, or Methods section.

n/a	Confirmed	
☐	☒	The exact sample size (*n*) for each experimental group/condition, given as a discrete number and unit of measurement
☐	☒	A statement on whether measurements were taken from distinct samples or whether the same sample was measured repeatedly
☐	☒	The statistical test(s) used AND whether they are one- or two-sided<br>*Only common tests should be described solely by name; describe more complex techniques in the Methods section.*
☐	☒	A description of all covariates tested
☐	☒	A description of any assumptions or corrections, such as tests of normality and adjustment for multiple comparisons
☐	☒	A full description of the statistical parameters including central tendency (e.g. means) or other basic estimates (e.g. regression coefficient) AND variation (e.g. standard deviation) or associated estimates of uncertainty (e.g. confidence intervals)
☐	☒	For null hypothesis testing, the test statistic (e.g. *F*, *t*, *r*) with confidence intervals, effect sizes, degrees of freedom and *P* value noted<br>*Give P values as exact values whenever suitable.*
☐	☐	For Bayesian analysis, information on the choice of priors and Markov chain Monte Carlo settings
☐	☒	For hierarchical and complex designs, identification of the appropriate level for tests and full reporting of outcomes
☐	☐	Estimates of effect sizes (e.g. Cohen's *d*, Pearson's *r*), indicating how they were calculated

Our web collection on statistics for biologists contains articles on many of the points above.

Software and code

Policy information about availability of computer code

Data collection	The MATLAB codes used to translate the raw sensor field data into light intensity (lux) corrected for field temperatures (degrees celsius), battery voltage and sensor calibration data are provided in the figshare depository (https://figshare.com/s/5958b330d9ae094db4c8).
Data analysis	The R code used for the repeated measures ANOVA is provided in the figshare depository (https://figshare.com/s/5958b330d9ae094db4c8).

For manuscripts utilizing custom algorithms or software that are central to the research but not yet described in published literature, software must be made available to editors and reviewers. We strongly encourage code deposition in a community repository (e.g. GitHub). See the Nature Research guidelines for submitting code & software for further information.

Data

Policy information about availability of data

All manuscripts must include a data availability statement. This statement should provide the following information, where applicable:

- Accession codes, unique identifiers, or web links for publicly available datasets
- A list of figures that have associated raw data
- A description of any restrictions on data availability

The datasets generated and/or analyzed during the current study are available on the figshare depository (https://figshare.com/s/5958b330d9ae094db4c8).

Field-specific reporting

Ecological, evolutionary & environmental sciences study design

All studies must disclose on these points even when the disclosure is negative.

Study description	This study aimed to test the solar refuge hypothesis for surviving Partula hyalina in the valleys of Tahiti. It involved designing, testing and calibrating a smart solar sensor at the University of Michigan, then applying the sensors in the field to aestivating Partula hyalina and forging predators to characterize their respective solar ecologies. We had three treatments: Partula hyalina leaf top, P. hyalina under leaf, and the predator Euglandina rosea). Field recordings were taken from each of the three categories (Partula hyalina leaf top, P. hyalina under leaf, and Euglandina rosea) over 8 days and light intensities were aggregated into their respective 10-minute time intervals from 9:30 hours to 16:00 hours.
Research sample	Two field populations of E. rosea and three of P. hyalina located in five northern valleys of Tahiti-Nui, the main Tahitian peninsula, were investigated in August 2017.
Sampling strategy	Each working day, we entered the study valley in the early morning between 8-9 a.m., prior to the appearance of the sun above the valley walls, and searched systematically for our respective target species. Euglandina rosea individuals were found foraging actively, either on the ground or climbing on vegetation, and they typically maintained this searching activity throughout the day. In contrast, Partula hyalina individuals were aestivating attached to the underside of leaves, and specimens typically remained in situ on the same leaf during the observation period. In 4/5 locations target snails were hard to find and our sample size was determined by the number of individuals we encountered on a given day. In the Tipaerui-Iti location, Partula hyalina were relatively abundant and the sample size was determined by the number of functioning sensors.
Data collection	The field data collection was performed by C. Bick, I.H. Lee and T. Coote. To track the solar ecology of each predator, a smart solar sensor was reversibly attached to the dorsal surface of each E. rosea shell using a nut and screw method. Aestivating Partula hyalina attach to the underside of leaves. Because our permit did not allow the direct attachment of light sensors to this endangered species, we deployed under-leaf sensors next to the aestivating snails using a nut/screw/magnets combination. we also recorded the ambient light intensity by attaching a sensor to the upper surface of the leaves harboring the aestivating specimens. Each working day, the data recording function of the smart sensors was activated before going into the field and was terminated after returning from the field, and the data were then retrieved via the sensors' wireless communication link. For each sensor, the recording start time, meaningful time of the measurement start time, meaningful measurement end time, and sensor recording end time were recorded to properly calibrate the time of the recorded samples. The received raw data in digital format were then translated to time and light intensity information using a MATLAB program and the calibration data specific for that sensor.
Timing and spatial scale	Light sensor recordings occurred from the time the rising sun cleared the valley walls (~9:30) until the setting sun fell back below them (~3:30pm). The spatial scale of each recording was on a single leaf for each aestivating Partula hyalina and within a few square meters for each foraging Euglandina rosea.
Data exclusions	Most aestivating P. hyalina (N=26/41) had two under leaf sensors bracketing the snails to record their immediate light environment (Figure 2b) and for these individuals we used the mean light intensity of the two sensors to compare to the other two categories.
Reproducibility	Individual sensors were used multiple times over the course of the field work on both species of interest. Three of the five study sites (Fautaua, Fautaua-Iti and Tipaerui-Iti) were sampled over two days of recording (non-adjacent days). Each study Partula hyalina was visually marked to avoid resampling.
Randomization	Recordings from each of the three categories (Partula hyalina leaf top, P. hyalina under leaf, and Euglandina rosea) over the 8 days of field recording were aggregated into their respective 10-minute time intervals from 9:30 hours to 16:00 hours and compared using a repeated measures analysis of variance (ANOVA).
Blinding	Blinding was not possible during data acquisition and most of the analyses, but within the three categories ((Partula hyalina leaf top, P. hyalina under leaf, and Euglandina rosea), data from multiple locations and days were analyzed collectively (Table 1; Fig. 3).

Did the study involve field work? ☒ Yes ☐ No

Field work, collection and transport

Field conditions	Low altitude tropical rain forest with mix of sunny and overcast conditions.
Location	The island of Tahiti - see Fig. 1a.
Access & import/export	Field work permits were obtained from the Delegation a la Recherche (de la Polynésie Française) - see the figshare depository (https://figshare.com/s/5958b330d9ae094db4c8). No specimens or tissues were removed from the field, only light sensor recording data.
Disturbance	The aestivating Partula hyalina were undisturbed during this study - the sensors were attached to the supporting leaves only.

Reporting for specific materials, systems and methods

We require information from authors about some types of materials, experimental systems and methods used in many studies. Here, indicate whether each material, system or method listed is relevant to your study. If you are not sure if a list item applies to your research, read the appropriate section before selecting a response.

Materials & experimental systems

n/a	Involved in the study
x	Antibodies
x	Eukaryotic cell lines
x	Palaeontology and archaeology
	x Animals and other organisms
x	Human research participants
x	Clinical data
x	Dual use research of concern

Methods

n/a	Involved in the study
x	ChIP-seq
x	Flow cytometry
x	MRI-based neuroimaging

Animals and other organisms

Policy information about studies involving animals; ARRIVE guidelines recommended for reporting animal research

Laboratory animals	The study did not involve laboratory animals.
Wild animals	The aestivating Partula hyalina were undisturbed during this study - the sensors were attached to the supporting leaves only. To track the solar ecology of each Euglandina rosea predator, a smart solar sensor was reversibly attached to the dorsal surface of each E. rosea shell using a nut and screw method. The nut (McMaster-Carr, Brass Hex Nut, narrow, 0-80 thread size) was glued (Loctite, Super Glue) directly on the predator's shell, and after allowing 10 mins for bonding, a sensor, pre-glued to a compatible screw (McMaster-Carr, 18-8 Stainless Steel Socket Head Screw 0-80 thread size, 1/16" long), was attached mechanically. Each predator was numerically labeled using nail polish and released at the exact spot it had been discovered. For the rest of the study period, each predator was visually tracked as it continued its foraging until mid-afternoon, when the sun descended below the valley walls, and the snails and sensors were recovered. These alien invasive predators were then euthanized by immersing in 95% ethanol.
Field-collected samples	To calibrate smart senors prior to field-deployment, field-collected specimens of Cepaea nemoralis were collected in Michigan by Mary Leys and made available to C. Bick and D. Ó Foighil. These were housed in modified aquaria (Supplementary Figure 8) at room temperature under a natural photoperiod in the Ó Foighil lab for one week and then released back into the wild.
Ethics oversight	University of Michigan Institutional Animal Care and Use Committee.

Note that full information on the approval of the study protocol must also be provided in the manuscript.
